# Death from Thomsonism

**Published:** 1842-05-14

**Authors:** 


*Deaths from Thomsonism.*—Dr. John Butterfield, of Lowell, Mass., reports, in the Boston Journal for May 11th, a case of erysipelas, 'in the onset not severe,' in which the patient was treated by a Thomsonian quack. He took capsicum, was put through a regular *course*, and finally steamed three times in twenty-four hours! In a state of coma with convulsions, he was handed over to the "regulars," and died after lingering a short time.

